# Basic Psychological Needs and Mental Health in Primary Care: A Self-Determination Theory Perspective

**DOI:** 10.3390/ijerph23080945

**Published:** 2026-07-23

**Authors:** Adam Neufeld, Savannah Roy

**Affiliations:** 1 Department of Family Medicine, Cumming School of Medicine, University of Calgary, 3330 Hospital Drive NW, Calgary, AB T2N 4N1, Canada; 2 Division of Behavioral and Organizational Sciences, Claremont Graduate University, 150 E Tenth St., Claremont, CA 91711, USA; savannah.carpenter@cgu.edu

**Keywords:** basic psychological needs, self-determination theory, resilience, coping, mental health, primary care

## Abstract

**Highlights:**

**Public health relevance—How does this work relate to a public health issue?**

- Mental health concerns are increasingly managed in primary care, yet healthcare systems often remain reactive, fragmented, and focused on symptom management.
- This study examines how satisfaction and frustration of basic psychological needs relate to resilience, vitality, coping, anxiety, and depression among adult primary care patients.

**Public health significance—Why is this work of significance to public health?**

- Findings support Self-Determination Theory by demonstrating that psychological need satisfaction is most strongly associated with well-being, whereas need frustration is most strongly associated with psychological distress in primary care.
- The results identify autonomy, competence, and relatedness as potentially scalable psychological resources that may strengthen prevention, coping, and mental health promotion.

**Public health implications—What are the key implications or messages for practitioners, policy makers and/or researchers in public health?**

- Incorporating psychological need-based perspectives into primary care may complement symptom-focused approaches and promote more holistic mental health care.
- Future research should examine how healthcare environments and clinical interactions support—or undermine—patients’ experiences of autonomy, competence, and relatedness.

**Abstract:**

Mental health concerns are increasingly encountered in primary care, yet clinical approaches often focus on symptom management rather than underlying psychological processes. Self-Determination Theory (SDT) proposes that psychological well-being depends on the satisfaction of three basic psychological needs—autonomy, competence, and relatedness—while their chronic frustration contributes to psychological distress. Despite extensive evidence linking these needs to mental health outcomes, their relevance in primary care remains underexplored. This single-center cross-sectional study examined associations between psychological need satisfaction and frustration and indicators of well-being and ill-being among adult primary care patients. A total of 380 patients (*M*_age_ = 47.81, *SD*_age_ = 15.42) completed validated measures of need satisfaction and frustration, resilience, vitality, coping strategies, anxiety, and depression. A dual-process model was examined, with hypotheses informed by SDT: higher need satisfaction associated with greater resilience, vitality, and adaptive coping (well-being), and higher need frustration associated with greater anxiety, depression, and maladaptive coping (ill-being). Although results revealed several significant cross-paths, need satisfaction was most strongly associated with positive outcomes and need frustration was most strongly associated with negative outcomes. The dual-process model did appear clear for coping outcomes, with need satisfaction uniquely predicting adaptive coping and need frustration uniquely predicting maladaptive coping. Findings highlight the potential value of incorporating psychological need-based perspectives into primary care mental health approaches. By recognizing autonomy, competence, and relatedness as foundational psychological resources, healthcare systems may develop more scalable strategies to promote mental well-being and prevent psychological distress in everyday clinical practice.

## 1. Introduction

### 1.1. Psychological Health and the Strain on Primary Care

Across Canada and globally, healthcare systems are increasingly strained by rising psychological distress and mental health disorders [1]. Depression, anxiety, and stress-related conditions now rank among the leading causes of disability worldwide, contributing substantially to the global disease burden due to their high prevalence and functional impact [2]. The World Health Organization estimates that approximately one in nine people globally—approximately 1 billion individuals—lives with a mental disorder, and suicide remains a leading cause of preventable death worldwide [3]. Beyond emotional suffering, these conditions affect daily functioning, productivity, physical health, and long-term mortality, with elevated risks for cardiovascular disease, diabetes, obesity, and earlier death [4,5,6].

Despite the scale of this challenge, mental health systems remain reactive and under-resourced. In Canada, wait times for psychiatric care often span months, and access to psychological services is uneven. Psychologists—among the most qualified professionals to assess and treat mental illness—are rarely covered by provincial health plans, rendering their services inaccessible to many individuals without private insurance or financial means. As a result, mental health is widely recognized as a societal priority yet remains structurally marginalized within healthcare delivery.

This gap places substantial pressure on family physicians, who increasingly function as the frontline providers for mental health care. In primary care settings, physicians must often assess psychological distress, initiate treatment, manage medications, coordinate referrals, and provide ongoing support within brief clinical encounters. Many physicians report feeling under-prepared or professionally isolated when navigating complex psychiatric needs, particularly when specialist access is limited [7,8]. The result is a system that frequently focuses on symptom management—often through pharmacologic treatment—while broader psychological mechanisms underlying mental health remain insufficiently addressed.

### 1.2. Psychological Needs and Mental Health

Self-Determination Theory (SDT) offers a well-established framework for understanding human motivation, development, and the psychological processes that support mental health and flourishing [9]. According to SDT, individuals possess three basic psychological needs: autonomy (the experience of volition and self-direction), competence (the experience of effectiveness and growth), and relatedness (the experience of connection and belonging). When these needs are supported within social environments, individuals tend to experience greater psychological well-being, vitality, and resilience [9]. Conversely, when these needs are chronically frustrated, individuals are more vulnerable to psychological distress, disengagement, and mental health problems [9].

A central insight from SDT is its dual-process model, which distinguishes between pathways to well-being and pathways to ill-being. Psychological need satisfaction promotes adaptive functioning, including positive affect, vitality, resilience, and constructive coping. In contrast, psychological need frustration—experiences of coercion, failure, rejection, or disconnection—predicts anxiety, depression, burnout, and maladaptive coping strategies. A growing body of research across psychology, education, and health contexts supports this model, demonstrating that need satisfaction and frustration represent distinct predictors of positive and negative psychological outcomes [10].

### 1.3. Dark Path: Need Frustration and Psychopathology

A growing body of clinical research suggests that need frustration may play an important role in the development and maintenance of psychopathology. Lower levels of need satisfaction and higher levels of need frustration have been associated with depressive disorders, anxiety symptoms, and broader psychological distress across diverse populations [11,12]. Beyond internalizing symptoms, need frustration has also been linked to maladaptive personality functioning and interpersonal disturbance [13]. Emerging work in clinical populations further suggests that diminished experiences of autonomy, competence, and relatedness may contribute to motivational and functional difficulties observed in severe mental illnesses such as schizophrenia [14]. Together, these findings suggest that basic psychological needs may represent a transdiagnostic mechanism underlying vulnerability to distress and psychopathology.

### 1.4. Bright Path: Need Satisfaction, Well-Being, and Recovery

Complementing this vulnerability pathway, need satisfaction appears to support the kinds of psychological resources that allow individuals to adapt effectively to stress and illness. Individuals who experience greater autonomy, competence, and relatedness in their daily lives tend to report higher vitality, resilience, adaptive coping, and overall well-being [15]. Research across both general and clinical populations indicates that higher levels of need satisfaction are associated with lower distress and greater well-being [11,16]. Evidence from large population studies further suggests that these associations are broadly consistent across demographic groups and across the lifespan, supporting SDT’s claim that psychological needs represent universal foundations of human well-being [17]. Together, these findings suggest that need satisfaction may represent a foundational psychological resource that helps individuals remain resilient in the face of adversity and avoid progression toward more persistent forms of distress.

### 1.5. Psychological Needs, Social Context, and Health

Although psychological needs appear to function as universal foundations of human well-being, individuals’ opportunities to experience autonomy, competence, and relatedness are shaped by their broader life circumstances. Socioeconomic conditions, access to resources, social relationships, and other structural determinants influence whether individuals experience their lives as self-directed, effective, and socially connected. Recent research applying SDT to the social determinants of health suggests that favorable social conditions may promote well-being in part by enabling need satisfaction. In contrast, disadvantage and instability can increase need frustration and associated distress [18].

Psychological needs may also shape how individuals cope with illness and engage in health-related behaviors. Meta-analytic research suggests that individuals who experience greater autonomy, competence, and relatedness are more likely to demonstrate sustained motivation for self-care, adaptive coping, and active engagement in managing health challenges [19]. Conversely, individuals experiencing chronic need frustration may struggle with disengagement, avoidance, or maladaptive coping strategies that can complicate recovery and disease management. In this way, psychological need experiences may influence not only mental health outcomes but also broader patterns of health behavior and adjustment to illness.

### 1.6. Psychological Needs in Primary Care Contexts

Primary care settings frequently encounter the consequences of unmet psychological and social needs. Patients presenting with anxiety, depression, chronic stress, or maladaptive coping (e.g., substance misuse) often describe experiences of feeling trapped in difficult circumstances, feeling ineffective in managing life demands, or feeling socially isolated—patterns widely documented in primary care and mental health research [20,21,22]. These experiences frequently arise in the context of common stressors, including financial insecurity, employment or caregiving pressures, chronic disease or pain, and major life transitions such as bereavement, illness diagnoses, or changes in work or family roles, all of which are known to shape psychological and physical health outcomes [18,23]. Emerging evidence from chronic disease populations further suggests that psychological need-supportive processes play an important role in shaping coping and self-management, including in such conditions as chronic pain [24]. Viewed through this lens, the psychological conditions described within SDT—autonomy, competence, and relatedness—may be highly relevant for understanding how patients experience and respond to mental health difficulties and broader health challenges in everyday clinical practice.

Yet despite extensive evidence linking psychological needs to mental health outcomes, these needs are rarely explicitly considered within clinical models of mental health care. Instead, clinical approaches often focus on symptom reduction without addressing the underlying motivational and relational conditions that shape psychological functioning. Integrating psychological need perspectives into primary care may therefore offer a complementary framework for understanding and addressing mental health challenges. Because primary care often serves as the first and most continuous point of contact for patients navigating both health concerns and life stressors, it provides a unique vantage point for understanding how psychological needs intersect with mental health, coping, and everyday functioning.

### 1.7. Study Purpose

Guided by SDT’s dual-process model of well-being and ill-being, the present study examines how psychological need satisfaction and need frustration relate to mental health outcomes among adult primary care patients. Specifically, we investigate whether need satisfaction is associated with indicators of well-being, including resilience, vitality, and adaptive coping, and whether need frustration is associated with indicators of ill-being, including anxiety, depression, and maladaptive coping (with cross-paths also examined). By examining these relationships within a community-based primary care population, this study aims to extend SDT research into a clinical context where psychological distress and health-related coping challenges are frequently encountered.

## 2. Materials and Methods

### 2.1. Participants and Procedure

A total of 500 patients from an urban community medical clinic in Calgary, Alberta, were invited to complete an anonymous online survey, which remained open for eight weeks between July and September 2025. Eligible participants were between 18 and 75 years old, cognitively fit, able to read and understand basic English, and had an active email address on file. Recruitment occurred through a combination of passive and active strategies: informational flyers were posted throughout the clinic, and patients were either verbally invited by their physician (author AN) during a medical visit or expressed spontaneous interest after seeing the flyers.

Interested patients received an email from a third-party address containing an ethics-approved consent form and a secure Qualtrics survey link. Four hundred and thirty-six participants completed the online survey. Participation was voluntary, confidential, and anonymous. No follow-up reminders were issued, and clinical care was unaffected by participation status.

This recruitment approach was approved by the University of Calgary’s Conjoint Health Research Ethics Board and aligned with national ethical guidelines [25], which supports physician-led recruitment provided participation is voluntary, informed, and does not affect clinical care.

### 2.2. Measures

Demographics. Participants self-reported their age (continuous), gender, marital status, education level, household income, and ethnicity (all categorical), and financial satisfaction (continuous; rated on a 0–10 scale). They also indicated the importance of religion in their daily life, current mental health status, recent help-seeking behavior, and whether they were taking psychiatric medications (all presented as categorical variables).

Basic Psychological Needs. Psychological need satisfaction and frustration were measured using the 18-item Basic Psychological Need Satisfaction and Frustration Scale (BPNSFS; general version). Nine items assessed satisfaction (e.g., “I feel capable at what I do”; “I feel a sense of choice and freedom in the things I undertake”) and nine assessed need frustration (e.g., “I feel like a failure because of the mistakes I make”; “My daily activities feel like a chain of obligations”). Responses were rated on a 7-point Likert scale (1 = not at all true to 7 = very true). Several items were reverse-coded to align with their respective constructs. Subscale scores were computed by averaging relevant items, with higher scores indicating greater need satisfaction or frustration. Internal reliability was strong for both subscales (satisfaction: α = 0.88; frustration: α = 0.86).

Resilience. Resilience was assessed using the 10-item Connor-Davidson Resilience Scale (CD-RISC-10), which evaluates the ability to cope with stress and adversity over the past month. Items were rated on a 5-point scale (0 = not true at all to 4 = true nearly all the time). Example items include: “I am able to adapt when changes occur” and “I believe I can achieve my goals, even if there are obstacles.” Mean scores were computed with higher scores indicating greater psychological resilience (α = 0.89).

Subjective Vitality. Subjective vitality was assessed using a 2-item version of the Subjective Vitality Scale (SVS), with participants rating statements on a 5-point scale (1 = strongly disagree to 5 = strongly agree). Items reflect one’s sense of aliveness and energy, such as “I have a lot of positive energy and initiative.” Mean scores were computed with higher scores indicating greater vitality (α = 0.67).

Coping. Coping was assessed using the 28-item Brief COPE inventory. To improve survey engagement and accuracy, participants were first asked to consider and briefly describe a recent stressful experience and then respond to the scale items based on how they coped with that situation. Items were rated on a 4-point scale (1 = “I haven’t been doing this at all” to 4 = “I’ve been doing this a lot”). Following established theory and prior literature, coping strategies were grouped into two composite domains: adaptive (e.g., active coping, planning, positive reframing, acceptance, emotional and instrumental support) and maladaptive (e.g., self-distraction, denial, substance use, behavioral disengagement, self-blame). Mean scores were computed for each domain. This grouping followed established conceptual models rather than factor-analytic structure, consistent with prior coping research. In the present sample, reliability was sufficient for both domains (α = 0.88 for adaptive coping; α = 0.75 for maladaptive coping).

Depression. Depressive symptoms were measured using the 9-item Patient Health Questionnaire (PHQ-9), with items rated on a 4-point scale (0 = not at all to 3 = nearly every day). Items captured core depressive symptoms over the past two weeks, including “Little interest or pleasure in doing things” and “Feeling down, depressed, or hopeless.” Total scores ranged from 0 to 27, with higher scores indicating greater depressive symptom burden (α = 0.89).

Anxiety. Anxiety was measured using the 7-item Generalized Anxiety Disorder scale (GAD-7), which assesses common symptoms of generalized anxiety over the past two weeks (e.g., “Feeling nervous, anxious, or on edge”; “Not being able to stop or control worrying”). Items were rated on the same scale as the PHQ-9. Total scores ranged from 0 to 21, with higher scores indicating more anxiety symptoms (α = 0.91).

### 2.3. Data Analysis

All data were collected via Qualtrics and analyzed using R Studio (Version 4.5.3). Participants were removed if they were missing more than 10% of any measure (basic psychological needs: *n* = 22; resilience: *n* = 2; depression: *n* = 12; anxiety: *n* = 5; coping: *n* = 10), resulting in 385 participants. All other missing data were handled using Full Information Maximum Likelihood, allowing all available observations to contribute to parameter estimation under the assumption that the data were missing at random. Multivariate outliers were then examined using Mahalanobis distance (α = 0.001), with 5 outliers detected and removed (list-wise deleted), adjusting the final sample size to 380.

Descriptive statistics and bivariate correlations were computed for all study variables (see Table 1). Composite scores were calculated using standard procedures, including reverse-coding applicable BPNSFS items to obtain distinct need-satisfaction and need-frustration scores. See Table 2 for information regarding variable item and range information. Cronbach’s alpha coefficients ranged from 0.75 to 0.91 for all variables, indicating strong internal consistency, except for vitality at 0.67. This slightly lower value is not surprising, as vitality only has two items, thus contributing to a lower alpha value [26].

Multivariate normality was assessed using Mardia’s tests of skewness and kurtosis, the overall MVN test, and univariate Anderson–Darling tests. Results indicated that the data were not multivariate normal, with Mardia’s skewness and kurtosis, as well as the overall MVN test, all significant. Most individual variables also violated univariate normality, apart from adaptive coping, which appeared approximately normal. Given these deviations, robust maximum likelihood was employed, which provides robust standard errors, a scaled chi-square statistic, and parameter estimates that remain consistent under violations of multivariate normality.

Analyses were conducted in RStudio using the sem() function from the lavaan package (version 0.6.21). Because all variables were modeled as observed variables, the analyses constitute path analysis within a structural equation modeling (SEM) framework rather than latent variable SEM. The structural path model was specified a priori based on the dual-process model derived from Self-Determination Theory. Need satisfaction and need frustration served as the primary exogenous predictors. Consistent with the dual-process framework, need satisfaction was hypothesized to predict resilience, subjective vitality, and adaptive coping, whereas need frustration was hypothesized to predict depression, anxiety, and maladaptive coping. To provide a more stringent test of the dual-process model, cross-paths were also estimated such that both need satisfaction and need frustration were entered simultaneously as predictors of all outcome variables.

Need satisfaction, need frustration, and the demographic covariates were treated as exogenous observed variables. Consistent with the default handling of exogenous variables in lavaan (fixed.x = TRUE), their observed variances and covariances were incorporated into model estimation. Demographic variables identified as significant predictors in a saturated model were retained as covariates for the relevant endogenous variables. Specifically, gender (man vs. woman), and marital status (common law vs. single) were included as covariates for resilience; gender (man vs. woman), religious affiliation (somewhat and very important vs. not at all important), and age were included as covariates for adaptive coping; education level (lower than high school vs. college/university or higher) was included as a covariate for depression; marital status (divorced vs. single), education level (high school vs. college/university or higher), and age were included as covariates for anxiety; and age and the interaction between need frustration and age were included as covariates for maladaptive coping. Residual covariances among endogenous variables were not estimated.

## 3. Results

### 3.1. Participant Characteristics

Of the final sample, 43.6% identified as men, 55.6% as women, and less than 1% identified as non-binary or preferred not to disclose. The majority of participants (82.1%) identified as White, 0.5% as Black or African American, 2.1% as Latino or Hispanic, and 4.8% reported more than one ethnicity or race. Regarding marital status, 22.6% reported being single, 12.7% common law, 51.4% married, 6.8% divorced, 3.1% widowed, and 3.1% selected “other”.

For education, 2.9% had less than a high school, 20.0% had completed high school, and 76.9% reported college/university or higher. Annual household income was distributed as follows: 14.3% earned $49,000 or less, 30.9% earned $50,000–$99,000, and 53.8% earned $100,000 or more. Participants also rated their financial situation relative to that of other Canadians on a 0–10 scale (0 = Much Worse, 10 = Much Better), reporting an average score of *M* = 6.39, *SD* = 2.27, suggesting that participants perceived their income as somewhat better than that of others.

When asked about the importance of religion in daily life, 56.9% indicated it was not at all important, 26.0% somewhat important, and 16.9% very important. Regarding mental health, 27.0% reported seeking professional help in the past three months, 26.5% were currently taking medication for mental health challenges, and 36.6% believed they were currently experiencing mental health challenges; in each case, a small proportion (<2%) preferred not to disclose.

### 3.2. Associations Between Psychological Needs and Mental Health Outcomes

Model fit was examined by obtaining global model fit indices with cutoffs specified by Hu & Bentler [27]. This model returned strong fit, χ^2^ (42) = 67.47, *p* = 0.008, SRMR = 0.03, Robust CFI = 0.99, Robust TLI = 0.97, Robust RMSEA = 0.04.

**Figure 1:**
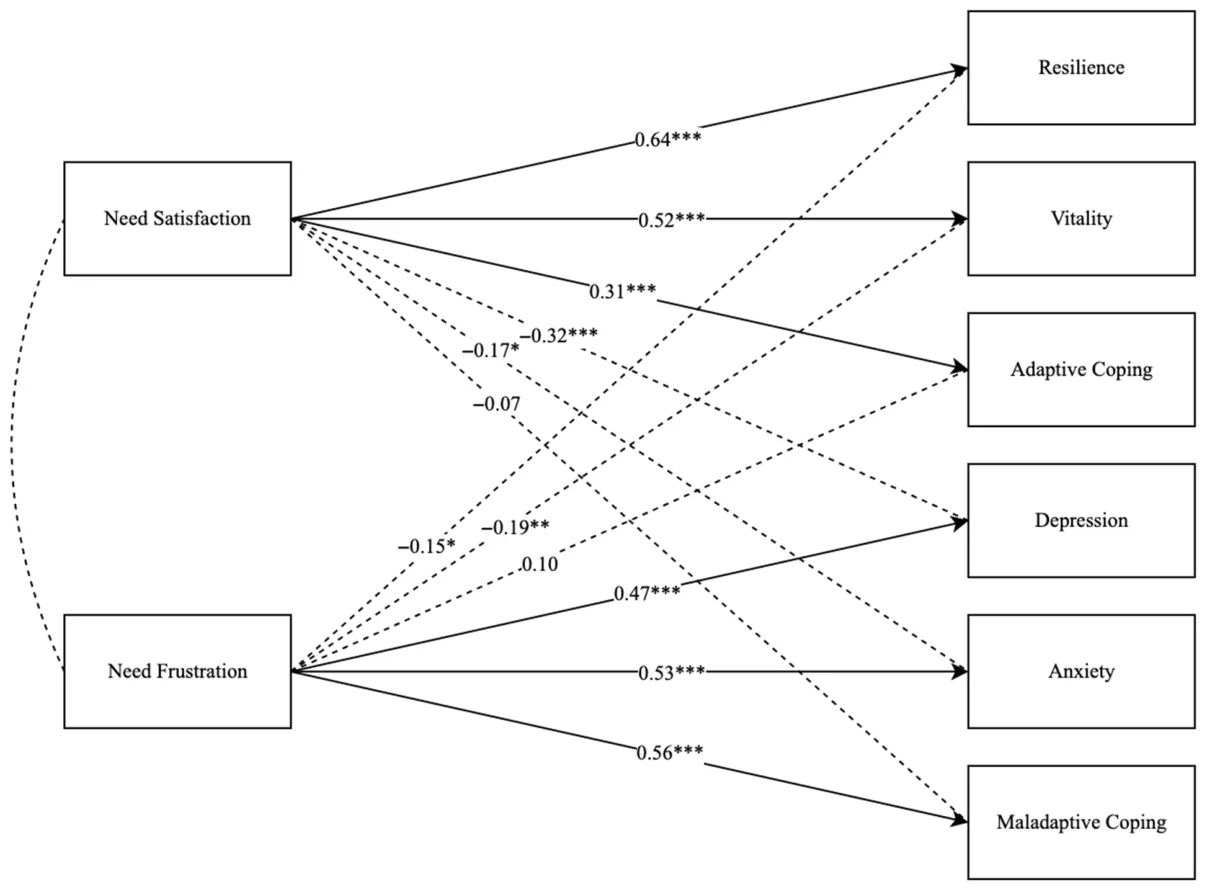
Statistical Model. Note. Values represent standardized path coefficients (β). Solid lines represent hypothesized paths, whereas dashed lines represent estimated cross-paths. *p* < 0.05 *, *p* < 0.01 **, *p* < 0.001 ***. Covariates and the interaction between need frustration and age predicting maladaptive coping are omitted for clarity. Need satisfaction and need frustration were treated as correlated exogenous variables.

Regression analyses examined associations between psychological need satisfaction, need frustration, and indicators of well-being and ill-being. Resilience was positively associated with need satisfaction (β = 0.64, *p* < 0.001) and negatively associated with need frustration (β = −0.15, *p* = 0.015). Adaptive coping was positively associated with need satisfaction (β = 0.31, *p* < 0.001) but not significantly related to need frustration. Vitality was positively associated with need satisfaction (β = 0.52, *p* < 0.001) and negatively associated with need frustration (β = −0.19, *p* = 0.004). Anxiety was positively associated with need frustration (β = 0.53, *p* < 0.001) and negatively associated with need satisfaction (β = −0.17, *p* = 0.010). Depression was positively associated with need frustration (β = 0.47, *p* < 0.001) and negatively associated with need satisfaction (β = −0.32, *p* < 0.001). Maladaptive coping was positively associated with need frustration (β = 0.56, *p* < 0.001) but not significantly related to need satisfaction. See Figure 1 for the statistical model. 

### 3.3. Demographic Analyses

All categorical demographic variables were dummy coded prior to analysis, with one category omitted as the reference group in each case. Ethnicity was not included in the analyses because the sample sizes for all groups, except White, were too small to allow reliable estimation. Age was mean-centered to facilitate interpretation and to reduce potential multicollinearity. Interaction terms were created between key psychological predictors (need satisfaction and need frustration) and demographic variables that demonstrated significant main effects in an initial saturated model. Any demographic predictors and interaction terms that were no longer statistically significant were removed, resulting in a more parsimonious final model.

Several demographic variables demonstrated significant direct effects on study outcomes. Individuals reporting a common law (vs. single) marital status exhibited higher resilience, whereas men (vs. women) exhibited lower resilience. In terms of coping, men (vs. women) and individuals who reported religion as somewhat or very important (vs. not at all important) demonstrated higher levels of adaptive coping. Age was negatively associated with both adaptive and maladaptive coping, indicating that coping behaviors decreased as age increased in the current sample.

With respect to mental health outcomes, individuals with less than a high school education (vs. college/university or higher) reported lower depressive symptoms. Additionally, those who were divorced (vs. single) and those with a high school education (vs. college/university or higher) reported lower anxiety. Age was also negatively associated with anxiety, suggesting that anxiety decreases as age increases in this sample.

The only demographic variable with a significant interaction was age on the relationship between need frustration and maladaptive coping, β = −0.10, *p* = 0.004. Simple slopes analyses indicated that the relationship between need frustration and maladaptive coping was significant at low, mean, and high levels of age. At one standard deviation below the mean age, need frustration was positively associated with maladaptive coping, *b* = 0.28, *SE* = 0.02, *z* = 15.27, *p* < 0.001. At the mean level of age, the association remained significant, *b* = 0.23, *SE* = 0.02, *z* = 15.29, *p* < 0.001. At one standard deviation above the mean age, the association also remained significant but was weaker, *b* = 0.18, *SE* = 0.02, *z* = 7.37, *p* < 0.001. These findings indicate that although greater need frustration was associated with greater maladaptive coping across all ages, this association was strongest among younger participants and progressively weaker among older participants.

## 4. Discussion

Guided by SDT’s dual-process model, this study examined how psychological need satisfaction and need frustration relate to well-being and ill-being among adults in a primary care population. Two primary patterns emerged. First, although need satisfaction and need frustration were each associated with both positive and negative outcomes, each construct demonstrated its strongest associations with its theoretically corresponding outcomes. Specifically, need satisfaction was more strongly associated with indicators of well-being (i.e., resilience, subjective vitality, and adaptive coping), whereas need frustration was more strongly associated with indicators of ill-being (i.e., depression, anxiety, and maladaptive coping). These findings suggest that need satisfaction and need frustration are not simply opposite ends of a single continuum but instead represent related yet distinct constructs that contribute differentially to psychological functioning.

Second, coping outcomes provided the clearest support for the dual-process model. Need satisfaction uniquely predicted adaptive coping but was not significantly associated with maladaptive coping after accounting for need frustration. Conversely, need frustration uniquely predicted maladaptive coping but was not significantly associated with adaptive coping after accounting for need satisfaction. This pattern indicates that adaptive and maladaptive coping are differentially associated with need satisfaction and need frustration, providing particularly strong support for the dual-process framework for coping processes.

These results are consistent with prior SDT research [9,11,28] and extend this work into a real-world primary care context, where individuals often present with complex and overlapping stressors. While SDT has been widely studied in educational and organizational settings, fewer studies have examined how psychological needs operate within everyday healthcare environments. The present findings suggest that these processes remain highly relevant in clinical populations and may help contextualize patterns of adaptation and distress observed in primary care.

Beyond these overall associations, the findings also highlight the importance of demographic and social context. Age moderated the relationship between need frustration and maladaptive coping, with younger individuals showing stronger associations, suggesting that younger patients may be particularly vulnerable when their needs are not met. These patterns align with broader research on the social determinants of health, which demonstrates that structural conditions shape mental health in part by constraining or supporting opportunities to experience autonomy, competence, and relatedness [18]. From this perspective, need satisfaction and frustration may represent psychological processes that warrant investigation as potential mechanisms in future longitudinal and experimental research.

The results also align with transdiagnostic perspectives, which emphasize shared psychological processes underlying diverse forms of distress. Within this framework, need frustration may represent one pathway associated with vulnerability, while need satisfaction may be associated with resilience and recovery. Clinically, this interpretation is consistent with evidence that individuals experiencing distress often report diminished perceived control, reduced self-efficacy, and social disconnection—experiences that closely parallel need frustration [29,30].

Importantly, this perspective complements rather than replaces existing clinical approaches to mental health care. While pharmacological treatments and specialist referrals remain essential, attention to psychological needs may offer a broader lens for understanding patient motivation, engagement, and recovery. Interventions that support patients in identifying meaningful goals, building competence in managing life challenges, and strengthening supportive relationships may represent important pathways toward improved well-being [31]. Because autonomy, competence, and relatedness represent universal psychological needs, SDT offers a theoretically grounded and scalable framework that can be integrated into routine clinical practice while also linking individual mental health outcomes to broader social conditions and determinants of health.

## 5. Limitations

Several limitations should be considered when interpreting these findings. First, the cross-sectional design prevents causal conclusions regarding the direction of associations between psychological needs and mental health outcomes. Longitudinal research is needed to examine how changes in need satisfaction and frustration influence mental health over time. Second, all measures relied on self-report, which may introduce shared method variance. Third, the study was conducted within a single primary care clinic, which may limit generalizability to other healthcare settings, especially given this sample was higher income (53.8% with an income of $100,000 or more), primarily White (82.1%), and highly educated (76.9% with college/university or higher education). Although physician-led recruitment was approved by the institutional research ethics board and participation was voluntary, anonymous, and unrelated to clinical care, some participants were verbally invited by their treating physician, who was also a study author. This recruitment strategy may have introduced selection bias or social desirability bias, which should also be considered when interpreting the findings.

## 6. Future Directions

Future research may further explore how healthcare environments influence psychological need experiences among patients. In particular, relational aspects of care, such as autonomy-supportive communication and patient-centered interactions, may play an important role in supporting patients’ psychological needs within clinical encounters. Longitudinal and intervention studies examining these processes may help clarify how healthcare systems can more effectively support well-being alongside traditional medical care. Future research might also explore additional outcomes within the dual-process framework to better understand how need satisfaction and need frustration should be conceptualized and applied, recognizing that their associations with outcomes may be shared for some domains but distinct for others.

## 7. Conclusions

Mental health concerns represent a growing challenge within primary care, yet clinical approaches often emphasize symptom management without fully addressing the conditions that support psychological functioning. The present findings suggest that experiences of autonomy, competence, and relatedness are closely linked to both well-being and vulnerability to ill-being in this context. These results support the relevance of SDT as a framework for understanding patient experiences in primary care and highlight the potential value of integrating need-based perspectives into clinical care. By supporting patients’ sense of agency, effectiveness, and connection, primary care may play an important role in promoting well-being alongside traditional approaches to treatment.

## Figures and Tables

**Table 1:** Means, Standard Deviations, and Correlations.

Variable	*M*	*SD*	1	2	3	4	5	6	7	8	9
1. Age	47.81	15.42									
2. Subjective Financial	6.39	2.27	0.06								
3. Need Satisfaction	5.45	0.97	0.12 *	0.43 **							
4. Resilience	2.75	0.67	0.13 *	0.35 **	0.77 **						
5. Vitality	3.14	0.93	0.12 *	0.33 **	0.67 **	0.62 **					
6. Adaptive Coping	2.42	0.60	−0.15 **	0.02	0.26 **	0.24 **	0.22 **				
7. Need Frustration	2.75	1.11	−0.20 **	−0.39 **	−0.78 **	−0.66 **	−0.59 **	−0.14 **			
8. Depression	6.87	5.77	−0.22 **	−0.37 **	−0.68 **	−0.59 **	−0.70 **	−0.09	0.71 **		
9. Anxiety	5.82	5.20	−0.31 **	−0.30 **	−0.58 **	−0.53 **	−0.55 **	0.04	0.67 **	0.77 **	
10. Maladaptive Coping	1.71	0.43	−0.28 **	−0.32 **	−0.51 **	−0.44 **	−0.44 **	0.20 **	0.64 **	0.68 **	0.69 **

Note. *p* < 0.05 *, *p* < 0.01 **.

**Table 2:** Variable Item and Range Information.

Variable	Number of Items	Response Set Min|Max	Theoretical Range Min|Max	Observed Range Min|Max	Scoring Metric
1. Age	1	18|75	18|75	18|75	Raw Score
2. Subjective Financial	1	0|10	0|10	0|10	Raw Score
3. Need Satisfaction	9	1|7	1|7	2.22|7	Mean
4. Resilience	10	0|4	0|4	0.5|4	Mean
5. Vitality	2	1|5	1|5	1|5	Mean
6. Adaptive Coping	14	1|4	1|4	1|3.94	Mean
7. Need Frustration	9	1|7	1|7	1|6.33	Mean
8. Depression	9	0|3	0|27	0|27	Total Score
9. Anxiety	7	0|3	0|21	0|21	Total Score
10. Maladaptive Coping	14	1|4	1|4	1|3.2	Mean

Note. Observed ranges reflect the scoring metric used in the analyses.

## Data Availability

The raw data supporting the conclusions of this article will be made available by the authors on request.

## References

[1] Wu Y., Wang L., Tao M., Cao H., Yuan H., Ye M., Chen X., Wang K., Zhu C. (2023). Changing trends in the global burden of mental disorders from 1990 to 2019 and predicted levels in 25 years. Epidemiol. Psychiatr. Sci..

[2] Charlson F., van Ommeren M., Flaxman A., Cornett J., Whiteford H., Saxena S. (2019). New WHO prevalence estimates of mental disorders in conflict settings: A systematic review and meta-analysis. Lancet.

[3] Arensman E., Scott V., De Leo D., Pirkis J. (2020). Suicide and Suicide Prevention from a Global Perspective. Crisis.

[4] Correll C.U., Solmi M., Veronese N., Bortolato B., Rosson S., Santonastaso P., Thapa-Chhetri N., Fornaro M., Gallicchio D., Collantoni E. (2017). Prevalence, incidence and mortality from cardiovascular disease in patients with pooled and specific severe mental illness: A large-scale meta-analysis of 3,211,768 patients and 113,383,368 controls. World Psychiatry.

[5] Holt R.I.G. (2019). The Management of Obesity in People with Severe Mental Illness: An Unresolved Conundrum. Psychother. Psychosom..

[6] Ranning A., Benros M.E., Thorup A.A.E., Davidsen K.A., Hjorthøj C., Nordentoft M., Laursen T.M., Sørensen H. (2020). Morbidity and Mortality in the Children and Young Adult Offspring of Parents with Schizophrenia or Affective Disorders—A Nationwide Register-Based Cohort Study in 2 Million Individuals. Schizophr. Bull..

[7] Loeb D.F., Bayliss E.A., Candrian C., DeGruy F.V., Binswanger I.A. (2016). Primary care providers’ experiences caring for complex patients in primary care: A qualitative study. BMC Fam. Pract..

[8] Neufeld A., Rahman A., Orakwue-Ononye N. (2023). Community Physician Perceptions of Managing Complex Child and Adolescent Psychiatric Patients: A Self-Determination Theory Perspective. J. Can. Acad. Child Adolesc. Psychiatry.

[9] Ryan R.M., Deci E.L. (2017). Self-Determination Theory: Basic Psychological Needs in Motivation, Development, and Wellness.

[10] Donald J.N., Bradshaw E.L., Conigrave J.H., Parker P.D., Byatt L.L., Noetel M., Ryan R.M. (2021). Paths to the Light and Dark Sides of Human Nature: A Meta-AnalyticReview of the Prosocial Benefits of Autonomy and the Antisocial Costs of Control. Psychol. Bull..

[11] Chen B., Vansteenkiste M., Beyers W., Boone L., Deci E.L., Van der Kaap-Deeder J., Duriez B., Lens W., Matos L., Mouratidis A. (2015). Basic psychological need satisfaction, need frustration, and need strength across four cultures. Motiv. Emot..

[12] Campbell R., Boone L., Vansteenkiste M., Soenens B. (2018). Psychological need frustration as a transdiagnostic process in associations of self-critical perfectionism with depressive symptoms and eating pathology. J. Clin. Psychol..

[13] Bartholomew K.J., Ntoumanis N., Ryan R.M., Bosch J.A., Thogersen-Ntoumani C. (2011). Self-Determination Theory and Diminished Functioning: The Role of Interpersonal Control and Psychological Need Thwarting. Personal. Soc. Psychol. Bull..

[14] Breitborde N.J.K., Kleinlein P., Srihari V.H. (2012). Self-determination and first-episode psychosis: Associations with symptomatology, social and vocational functioning, and quality of life. Schizophr. Res..

[15] Lépine F., Busque-Carrier M., Le Corff Y. (2025). The Mediating Role of Psychological Needs in the Association Between Work Values and Well-Being Indicators. Career Dev. Q..

[16] Milyavskaya M., Koestner R. (2011). Psychological needs, motivation, and well-being: A test of self-determination theory across multiple domains. Pers. Individ. Differ..

[17] Lataster J., Reijnders J., Janssens M., Simons M., Peeters S., Jacobs N. (2022). Basic Psychological Need Satisfaction and Well-Being Across Age: A Cross-Sectional General Population Study among 1709 Dutch Speaking Adults. J. Happiness Stud..

[18] Bradshaw E.L., DeHaan C.R., Neufeld A., Diaz-Castillo S., Ryan R.M. (2026). A self-determination theory approach to the social determinants of health and psychological wellbeing. Psychol. Health Med..

[19] Ntoumanis N., Ng J.Y.Y., Prestwich A., Quested E., Hancox J.E., Thøgersen-Ntoumani C., Deci E.L., Ryan R.M., Lonsdale C., Williams G.C. (2021). A meta-analysis of self-determination theory-informed intervention studies in the health domain: Effects on motivation, health behavior, physical, and psychological health. Health Psychol. Rev..

[20] Mercer S.W., Guthrie B., Furler J., Watt G.C.M., Hart J.T. (2012). Multimorbidity and the inverse care law in primary care. BMJ.

[21] Barnett K., Mercer S.W., Norbury M., Watt G., Wyke S., Guthrie B. (2012). Epidemiology of multimorbidity and implications for health care, research, and medical education: A cross-sectional study. Lancet.

[22] Coventry P.A., Hudson J.L., Kontopantelis E., Archer J., Richards D.A., Gilbody S., Lovell K., Dickens C., Gask L., Waheed W. (2014). Characteristics of effective collaborative care for treatment of depression: A systematic review and meta-regression of 74 randomised controlled trials. PLoS ONE.

[23] Lund C., Brooke-Sumner C., Baingana F., Baron E.C., Breuer E., Chandra P., Haushofer J., Herrman H., Jordans M., Kieling C. (2018). Social determinants of mental disorders and the Sustainable Development Goals: A systematic review of reviews. Lancet Psychiatry.

[24] Stenberg N., Gillison F., Rodham K. (2022). How do peer support interventions for the self-management of chronic pain, support basic psychological needs? A systematic review and framework synthesis using self-determination theory. Patient Educ. Couns..

[25] Bibbins-Domingo K., Brubaker L., Curfman G. (2025). The 2024 Revision to the Declaration of Helsinki Modern Ethics for Medical Research. JAMA.

[26] Eisinga R., Grotenhuis M.T., Pelzer B. (2013). The reliability of a two-item scale: Pearson, Cronbach, or Spearman-Brown?. Int. J. Public Health.

[27] Hu L.T., Bentler P.M. (1999). Cutoff criteria for fit indexes in covariance structure analysis: Conventional criteria versus new alternatives. Struct. Equ. Model..

[28] Vansteenkiste M., Ryan R.M. (2013). On psychological growth and vulnerability: Basic psychological need satisfaction and need frustration as a unifying principle. J. Psychother. Integr..

[29] Gallagher M.W., Bentley K.H., Barlow D.H. (2014). Perceived Control and Vulnerability to Anxiety Disorders: A Meta-analytic Review. Cogn. Ther. Res..

[30] Holt-Lunstad J., Smith T.B., Baker M., Harris T., Stephenson D. (2015). Loneliness and Social Isolation as Risk Factors for Mortality: A Meta-Analytic Review. Perspect. Psychol. Sci..

[31] Neufeld A. (2025). Life Aspirations and Health in Canada: A Patient-Oriented Study. Can. J. Behav. Sci..

